# Supplementary material for: Splice-Junction-Based Mapping of Alternative Isoforms in the Human Proteome
Source: Cell Rep. Author manuscript; Available in PMC 2020 Jan 15. (PMC6961840; doi:10.1016/j.celrep.2019.11.026)
Supplement: 2 [file NIHMS1546469-supplement-2.zip › DF1/PXD006675/RightAtrium/RightAtrium_27_TTN_AESPPPEVPGGEK.pdf]

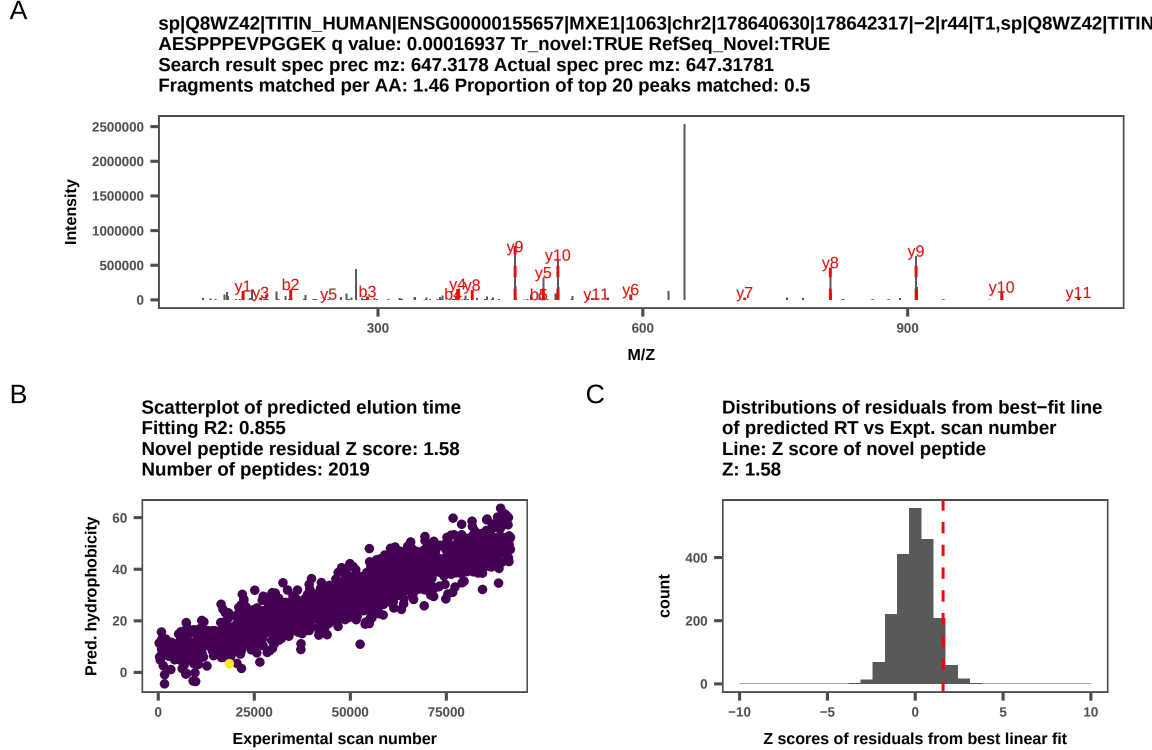
A
sp|Q8WZ42|TITIN_HUMAN|ENSG00000155657|MXE1|1063|chr2|178640630|178642317|−2|r44|T1,sp|Q8WZ42|TITIN
AESPPPEVPGGEK q value: 0.00016937 Tr_novel:TRUE RefSeq_Novel:TRUE
Search result spec prec mz: 647.3178 Actual spec prec mz: 647.31781
Fragments matched per AA: 1.46 Proportion of top 20 peaks matched: 0.5
Intensity
2500000
2000000
1500000
1000000
500000
0
y1
y3
b2
y5
b3
b4
y4
y8
y9
y5
y10
b5
y11
y6
y7
y8
y9
y10
y11
300
600
900
M/Z
B
Scatterplot of predicted elution time
Fitting R2: 0.855
Novel peptide residual Z score: 1.58
Number of peptides: 2019
Pred. hydrophobicity
60
40
20
0
0
25000
50000
75000
Experimental scan number
C
Distributions of residuals from best−fit line
of predicted RT vs Expt. scan number
Line: Z score of novel peptide
Z: 1.58
count
400
200
0
−10
−5
0
5
10
Z scores of residuals from best linear fit
